# Calcium (Ca2+) waves data calibration and analysis using image processing techniques

**DOI:** 10.1186/1471-2105-14-162

**Published:** 2013-05-16

**Authors:** Carlos Milovic, Carolina Oses, Manuel Villalón, Sergio Uribe, Carlos Lizama, Claudia Prieto, Marcelo E Andia, Pablo Irarrazaval, Cristian Tejos

**Affiliations:** 1 Department of Electrical Engineering, Pontificia Universidad Catolica de Chile, Santiago, Chile; 2 Biomedical Imaging Center, Pontificia Universidad Catolica de Chile, Santiago, Chile; 3 PixInsight Development Team, Pleiades Astrophoto S. L, Valencia, Spain; 4 Faculty of Biology, Pontificia Universidad Catolica de Chile, Santiago, Chile; 5 Radiology Department, School of Medicine, Pontificia Universidad Catolica de Chile, Santiago, Chile; 6 Department of Mathematics and Computation Science, Universidad de Santiago de Chile, Santiago, Chile; 7 Division of Imaging Sciences and Biomedical Engineering, King’s College London, London, UK

**Keywords:** Calcium wave velocity, Two-filter method, Automation

## Abstract

**Background:**

Calcium (Ca2+) propagates within tissues serving as an important information carrier. In particular, cilia beat frequency in oviduct cells is partially regulated by Ca2+ changes. Thus, measuring the calcium density and characterizing the traveling wave plays a key role in understanding biological phenomena. However, current methods to measure propagation velocities and other wave characteristics involve several manual or time-consuming procedures. This limits the amount of information that can be extracted, and the statistical quality of the analysis.

**Results:**

Our work provides a framework based on image processing procedures that enables a fast, automatic and robust characterization of data from two-filter fluorescence Ca2+ experiments. We calculate the mean velocity of the wave-front, and use theoretical models to extract meaningful parameters like wave amplitude, decay rate and time of excitation.

**Conclusions:**

Measurements done by different operators showed a high degree of reproducibility. This framework is also extended to a single filter fluorescence experiments, allowing higher sampling rates, and thus an increased accuracy in velocity measurements.

## Background

Ciliated cells in the oviduct play a crucial role in transportation mechanisms. They carry the ovule prior to its implantation in the womb at a velocity that is proportional to the cilia beat frequency. The frequency rate is regulated by a variety of signals [1,2], including calcium level changes. Calcium is emitted and transmitted to nearby cells, and thus propagating calcium waves are generated [2].

Mechanical stimuli of a ciliated cell can produce local changes, such as opening calcium channels of the plasmatic membrane, rising IP3 levels and releasing ATP. These signals produce an increase of calcium concentration, and a wave begins to propagate [3] along the tissue. The propagation itself is regulated by several variables, including free Ca2+, and the presence of other proteins or hormones.

Intra-cellular Ca2+ can be measured *in vitro* in oviduct tissues using spectral-fluorimetric systems. Derivations of the Fura-2 fluorosphor are commonly used, since they are permeable to the cellular membrane, and have a high Ca2+ specificity. This fluorosphor has an optimal absorption rate of photons at a wavelength around 510 nm. Unbounded, the optimal emission is close to 380 nm, whereas bounded to Ca2+ the optimal emission is close to 340 nm. Image acquisition is performed with optical microscopes, using two narrow band filters (centered at 340 and 380 nm). The propagation of the Ca2+ wave is registered by recording a sequence of images, interlacing those filters. The ratio between consecutive 340 and 380 nm filtered images retrieves an index of the intracellular Ca2+ concentration at a specific time instant [2].

Current methods to characterize *in vitro* propagation of the Ca2+ waves involve several manual steps. The wave is commonly generated by a mechanical stimulus. The point where that stimulus is performed is identified in the image sequence by visual inspection. Then, Ca2+ is measured at different spatial locations. Sampled spatial locations are manually defined. In [4], each cell is manually segmented and defined as sampled regions. Other approaches consider defining sampled regions such as squares and ellipses, distributed uniformly in a matrix shape, along linear trajectories from the stimulus origin, or other simple geometrical patterns available in commercial image processing software [5]. Calcium concentration is averaged inside each sampled region, and the obtained value is transcribed to a data-sheet for further analysis. The Ca2+ wave-font is identified by setting an arbitrary intensity threshold. Usually, it is a percentile-based growth with respect to the basal conditions. The propagation velocity is derived from the activation time and location of the identified wave-front. Propagation is sometimes assumed to be radially symmetric. For those cases, computed velocities (or activation times) are averaged when they belong to wave-fronts located at the same radial distances, yielding a small set of measured speeds. The entire process is mostly performed manually and may take several hours to complete.

This kind of procedures has several drawbacks and requirements. (i) They are tedious and involve large processing times. (ii) Since they involve manual arbitrary decisions, they lack good inter and intra observer reproducibility. (iii) Standard data sheet software has limited computing capabilities, and managing large quantities of data is not practical. (iv) Sampling the propagation speed at a few discrete locations retrieves little information to properly characterize the wave. More samples (distances and amount) and characterization parameters such as the maximum amplitude and decay ratio would be ideal to better describe the wave. (v) More samples and more complex calculations would improve statistical confidence of the experiments, but that would lead to impractical computation times. (vi) The natural decay of the fluorescence marker has to be corrected, and better noise management routines are needed, especially to determine the basal conditions of the experiment. (vii) Automatic routines should be used to align individual frames to correct for the movements that may affect the experiment.

In this paper we present a method that takes raw captured images and calibrates them to extract and characterize accurately the traveling wave. Two different approaches (global and local) are presented for data analysis. This is performed in a semi automatic way, reducing the processing time, and increasing the reproducibility of the experiment. Data is analyzed for the entire image, increasing statistical confidence, and giving global and local information of the phenomenon.

Additionally, we propose a method to extract velocities from images obtained with a single filter. This allows higher sampling rates and accuracy to determine the speed of the wave.

## Methods

**Figure 1:**
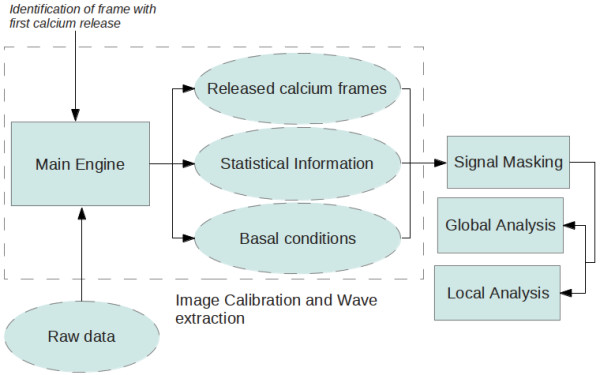
General work-flow.

Our proposed method has four stages: 1) Image Calibration and Wave Extraction, 2) Signal Masking, 3) Global Analysis, and 4) Local Analysis (Figure 1). Raw images captured by the CCD camera are passed to the Image Calibration and Wave Extraction stage. An optional intermediate signal masking process could be applied to speed up the analysis and to get more robust measurements. The results (with or without masking) are then analyzed either by a global or a local approach, leading to the final outputs: propagation velocity, wave decay, and wave amplitude.

### Image calibration and wave extraction

The purpose of this stage is to calibrate the data and isolate the Ca2+ that is released to the media using the raw images as input. The outputs are the basal conditions, statistical information of the set, and a sequence of images showing only the Ca2+ above the basal values. This change of concentration is associated with the passing wave, and hence it is important to accurately calibrate the whole set of frames, and to reduce any possible source of errors. To accomplish this, the user has to identify the frame where the wave propagation begins, and our software processes the frames according to the following steps:

#### Image registration

Since the Ca2+ wave is induced by a mechanical stimulation of a cell, minor motion may be introduced to the sample, and hence physical locations do not match exactly in all the frames. To correct that problem an automatic rigid alignment of the frames was implemented. We estimate the displacement between frames computing the Fourier Transform of each image, and calculating the difference of phase angles with respect to a reference frame. Since the images are captured with different filters, they do not have the same intensity mapping. A wavelet based pre-filtering is applied to the input data so that to obtain images with comparable intensities and reduced noise. This pre-filtering process consists on the following steps: first, the image is decomposed into wavelet layers, using the starlet transform [6] (formerly known as A Trous wavelets transform). Second, all wavelet layers are discarded, except for that corresponding to structures of 1px or 2px (user defined). Finally, this remaining layer is max-min rescaled to a normalized range.

**Figure 2:**
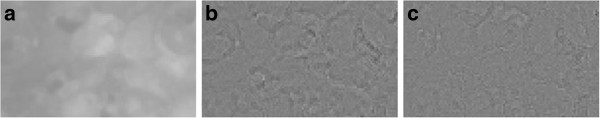
**Registration example using wavelet filtering. a**) Differences of misaligned frames at two different wavelengths. **b**) Differences of the first wavelet layer, same images (Std dev: 0.025). **c**) Differences of the first wavelet layer, after registration (Std dev: 0.021).

Figure 2 shows an example of this procedure. Figure a) shows the difference between a reference frame captured at 340 nm and a target frame at 380 nm. In this case the misregistration is about 3px in the vertical axis. Figure b) shows the difference between the rescaled layers of 1px of the same images. Figure c) is the new difference of frames, after registration with the Fourier phase correlation.

#### Calcium concentration calculation

The calcium concentration can be obtained after computing the ratio between the 340 and 380 nm filtered images. However, to estimate precisely such concentration an additional calibration function must be applied [7]. Since the 340:380 ratio shows an approximately linear relation with calcium concentration and we were focused in characterizing the wave propagation (and not absolute calcium concentrations), we performed our data calculations from the computed ratio without such calibration.

#### Marker decay correction

**Figure 3:**
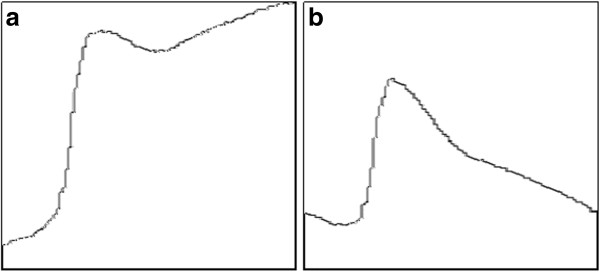
**Total calcium for each frame as a function of time.** (**a**) shows the 340:380 ratio computed from raw data, while (**b**) shows the corrected data by extrapolating with an exponential function derived from the basal conditions.

The fluorescent marker suffers a natural decay over time, following closely an exponential function [8]. If the decay time were similar for 340 and 380 nm, computing the ratio of both filtered images would correct that distortion. However, we empirically found that each wavelength shows a different decay time, generating a bias on the computed ratio. To correct this effect, we analyze the total flux of the computed ratio images and fit an exponential function to the frames captured before the mechanical stimulus. This function is extrapolated to the following frames. Since the fluorescence does not generally return to basal conditions in the captured sequence, data points are not fitted at the end, thus ensuring a better fit. Figure 3 illustrates an example of this procedure.

#### Basal conditions calculation

To detect the Ca2+ wave, we have to determine the basal conditions of the experiment. This is done analyzing the ratio images (with decay correction) prior to the mechanical stimulus. They are averaged to increase signal to noise ratio. To improve the results, a sigma reject algorithm is used to prevent noise or artifacts in the averaging process (pixel values outside a 2 sigma range from the mean value are discarded).

#### Wave estimation

If we assume that the Ca2+ wave consists only of molecules liberated to the media, without other sources, subtraction of the basal conditions image yields the desired result. Furthermore, since no negative values are expected, except from noise, these values are truncated to work only with positive data.

#### Noise reduction

We used a median filter to reduce noise [9], while preserving sharp and small-scale structures. The filter kernel should be smaller or equal to 5 × 5 pixels to achieve those goals.

#### Statistical data output

In this last step, statistical data of the wave is saved as additional images. This includes the mean, maximum, standard deviation values and time of the maximum for each pixel. Such information will be used for further analysis in later processing steps.

### Signal masking

Once we have a series of images containing the Ca2+ that is generated, we select an area of interest based on an intensity threshold. This speeds up the processes and avoids spurious data. We use images containing the statistical information from the previous step (e.g. the maximum value of the series and the average value for a given pixel) to choose the threshold as one standard deviation over the background intensity level. Although this value works for most of the cases, fine-tuning may be needed.

The result is a binary image, which is often contaminated with spurious small-scale structures associated with noise or artifacts, and with small signal voids in the excited cells. We solved these problems applying morphological closure and aperture binary operators [9]. The size of the operators (typically discs of 5 to 9 pixels in diameter) and order of execution yields different results, but running times are short, thus evaluation by visual inspection is affordable, almost in real time, testing a few configurations. This inspection is based mainly on preserving the shapes of the outer cells, without including background zones and excluding isolated sources outside the main connected group of cells.

Additionally, it is useful to restrict the maximum radius to bed of the samples to be inspected. Signal far away from the stimulus may corrupt calculations as the signal to noise ratio decreases with the distance.

### Global analysis

The purpose of the global analysis is to simplify the data, assuming radial symmetry of the propagation wave. To summarize the process, a pixel is defined as the propagation center and then sample regions are automatically defined on the images. Radial distance and calcium intensity are measured in those samples, and a single output image is generated. Each pixel on the output image displays the average intensity as a function of radial distance and time. This image will be further analyzed to measure the wave velocity and to fit decay models. The process is performed in four steps:

#### Propagation source determination

**Figure 4:**
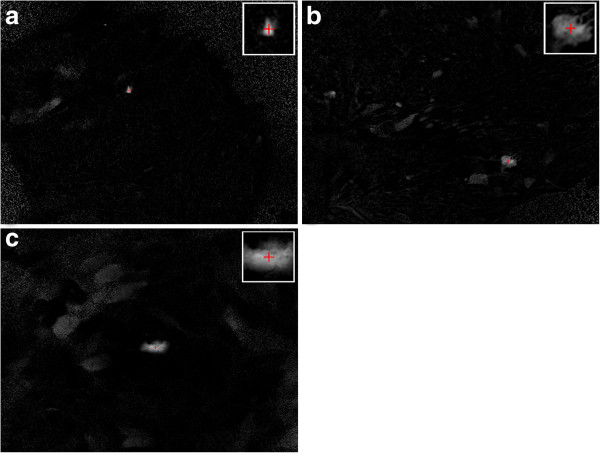
**Automatic determination of the source of the calcium wave.** Red cross shows the calculated center of mass of the brightest and larger structure. The sub-image at the top right corner of each image shows a magnified view at the calculated center of mass.

The user needs to define by visual inspection the frame in which the mechanical stimulus was performed and calcium begins to be released. The physical location of the stimulus is found automatically from this image. To do that, the image is first filtered with gray scale morphological operations [9] (erosion followed by dilation, both 3 × 3 pixels) so that to reduce noise and the result is linearly rescaled into a 0.0 to 1.0 range (normalized range). Pixels with intensity values below 0.2 are discarded (pixel values are replaced with zeroes). The center of mass is calculated by successive approximations or iterations. Each iteration consists of: (1) narrowing the region of calculation by half on each dimension and centered on the previously calculated center of mass. (2) Updating the center of mass. This process is done until at least a rectangle of 20 × 20 pixels is defined. This approach returns the center of mass of the biggest and brightest structure in the image, which should correspond to the center of the wave of interest (Figure 4). The process ensures that secondary calcium releases or noise do not affect the calculations. If this algorithm fails, the operator may manually correct those coordinates.

#### Sample generation

**Figure 5:**
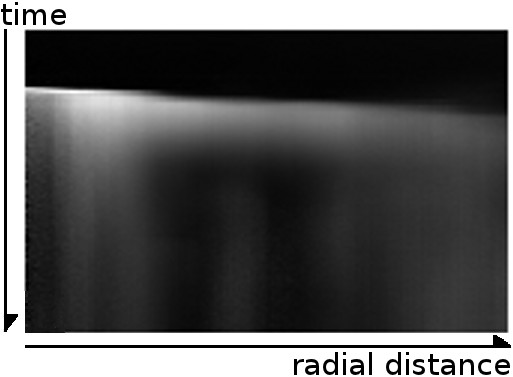
**Calcium concentration as function of radial distance and time.** Pixel intensities represent relative calcium concentration. Zero time and radius is set to the top left corner.

Since we are interested in radially symmetric wave propagations, we use concentric one pixel-width ring samples. Pixel values are averaged inside each ring. Each averaged ring is mapped as a new pixel in an output image, creating a graph of radial distance and time (Figure 5).

#### Linear global fit

Performing a linear regression fit to the simplified data can be done to quickly extract information. We characterize the wave propagation using three parameters: time of maximum intensity, time of maximum increase of intensity, and maximum intensity, each one as a factor of the radial distance from the mechanical stimulus.

•Time of Maximum Intensity: A linear regression of the radial distance versus time of the maximum intensity gives the mean propagation velocity of the maximum calcium concentration.

•Time of Maximum Increase of Intensity: Instead of looking for the maximum intensity in the image, we look at the maximum positive gradient. Since the calcium release to the medium is a fast process, individual pixels present a quick and large increase of intensity. Thus, a linear regression of the radial distance versus time of the maximum gradient gives another good measurement of the wave-front velocity.

•Maximum Intensity: Far from the mechanical stimulus, the maximum intensity that is reached can be well fitted by a linear decay function. Extrapolation of this function gives a measure of the maximum propagation radius.

#### Non-linear global fit

Our focus is now the time evolution of the calcium concentration for a given radial distance. For this, we used the CMinPack libraries (University of Chicago, 1999) [10], which consists of a Levenberg-Marquardt algorithm to fit non-linear models to data. To test the procedure we propose exponential, Eq. 1, and quadratic, Eq. 2, decays. Both models can be expressed in terms of three parameters, the wave maximum intensity (“A”), the time of activation (“t_0_”), and a decay rate (“τ”, tau).where H(t-t_0_) is the Heaviside function.

(1) $$f\left(t\right)=H_{\left(t-t_{0}\right)}\cdot A\cdot e^{\frac{-\left(t-t_{0}\right)}{\tau }}$$

(2) $$f\left(t\right)=\frac{H_{\left(t-t_{0}\right)}\cdot A\cdot \tau^{2}}{\tau^{2}+{\left(t-t_{0}\right)}^{2}}$$

**Figure 6:**
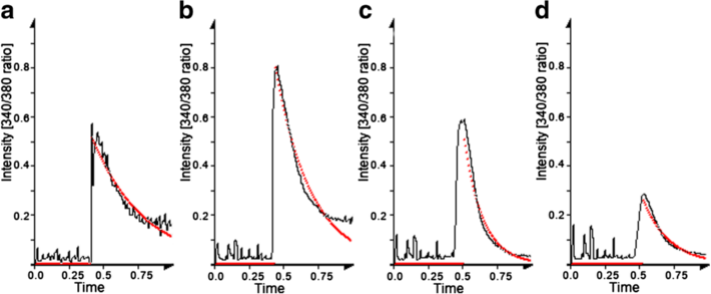
**Data evolution along time for random radial distances in a control example.** In red is shown the fitted exponential function. The measured distances are: **a**) 2 pixels, **b**) 23 pixels, **c**) 58 pixels and **d**) 95 pixels. The vertical axis shows relative calcium concentration (same scaling all the samples) and horizontal axis is the normalized time (0 is the beginning of acquisition, and 1 the end of acquisition).

We analyze these fitted variables as function of the radial distance (Figure 6) to achieve a deeper understanding of the wave propagation. In particular, a linear regression of the radial distance versus measured time of activation gives another measurement of the mean wave propagation speed.

### Local analysis

A non-linear fit can be performed to individual pixels, following the same procedure as in the Global Analysis. We use the Levenberg-Marquardt algorithm to find our three parameters pixel by pixel. This requires thousands of minimization operations. Masking the signal, as in section 2.2, greatly improves the efficiency of this procedure, since we analyze only those pixels that are part of the wave. The result gives a localized map of the variables that characterize the calcium concentration behavior in time.

### One filter wave propagation measurement

To calculate the calcium concentration, consecutive filtered images are acquired at two different wavelengths. If we are interested only in measuring the wave-front velocity, calculations can be done using only the 380 nm set. By using only one filter we are saving the time of the second exposure and that required to mechanically change the filters, allowing a higher sampling rate (from 0.8 to 2.8 frames per second in our setup). This yields to a more reliable estimation of velocity, since it is derived from the slope of the maximum gradient linear regression, in the global analysis.

The characterization procedures remain the same but without computing ratios.

The propagation velocity can be computed from both, the time at the maximum pixel intensity or the maximum increase in pixel values. As will be shown, the mean velocity of the calcium wave-front estimated from the maximum gradient is highly correlated to that found using two filters. Maximum intensity in the 380 nm images is not well correlated to wave maximum intensities found with two filters, thus estimations of the mean propagation velocity cannot be done from the time of maximum intensity.

### Implementation details

All stages were implemented in C++ language, as external modules for the image processing platform PixInsight (Pleiades Astrophoto S. L., Valencia, Spain). Raw images were stored in an 8-bit TIFF format. Loaded images were transformed to 32-bit floating-point samples, and outputs were stored as 32-bit FITS files to maintain numerical accuracy.

### Experimental setup

#### Primary cultures of oviductal ciliated cells

Epithelial cell cultures from rat oviduct were obtained as in [11]. Female adult rats *Sprague Dawley* (250–300 g) were used. Animals were kept under artificial illumination and controlled temperature (23–25°C) from 6:00 to 21:00 hours. Water and pelleted food were supplied *ad libitum*. Day 1 of the rat estrous cycle was assessed by the presence of a vaginal plug. On day 1, between 11:00 and 12:00 hours, animals were sacrificed by cervical dislocation after ketamine/Xilacine anesthesia i.p.

The genital tract was removed and oviducts were placed into Hanks’ solution (Sigma-Aldrich) at pH 7.4. The distal portion of the oviduct (i.e. the fimbria) was mechanically removed with fine forceps. The ciliated epithelium was extracted and transferred to a fresh culture medium. Epithelial explants were placed on 0.1% gelatin-treated coverslips of sealed chambers. The Rose Chambers were perfused with NHS medium containing 10% heat-inactivated horse serum. NHS had the following composition in mM: NaCl 137; KCl 5.09; Na2HPO4 1.14; KH2PO4 0.18; MgCl2 0.923; CaCl2 0.91; NaHCO3 4.07; glucose 21.5 and glutamine 0.2; supplemented with 1.0% vitamins, 1.0% essential amino acids, 1.0% non-essential amino acids, 1.0% pyruvate and antibiotics (0.2 mg/ml neomycin and 0.12 mg/ml penicillin), pH 7.2–7.4 at 37°C. Primary cultures were incubated at 37°C for 5–7 days. When this cell monolayer showed spontaneous ciliary activity, the chambers were opened and the cultures were washed three times with fresh Hanks’ solution before calcium measurements were performed.

These protocols were approved by the Animal Care and Ethics Committee of Pontificia Universidad Catolica de Chile.

#### Measurement of calcium waves

From manual estimations with a few samples, we observed some differences on the calcium wave propagation when the ciliated epithelium samples were exposed to different hormones [12]. Thus, we decided to perform three kinds of experiments: cultures as described in the previous section, and cultures exposed either to leptin or adiponectin.

Intracellular calcium levels were determined using a spectrofluorometric technique in primary cultures of oviductal epithelial cells as described in [4], with Fura 2-AM (acetoximetil ester, Invitrogen Molecular Probes, Inc. OR 97402–9132, USA). Intracellular calcium wave propagation velocity was measured in ciliated cell cultures previously incubated with different concentrations of leptin (Recombinant Rat leptin, R&D Systems, Inc., Minneapolis, MN 55413 USA), and adiponectin (Acrp30, R&D System, Inc. Minneapolis, MN 55413 USA) for a period of 48 hours.

Calcium waves were generated with a mechanical stimulus, which consisted in a borosilicate glass micropipette (Harvard Apparatus Lts, UK) for patch-clamp, mounted on a micromanipulator. The micropipette descended gradually to touch the surface of a cell, generating an increase of calcium, which spreads to neighboring cells. Video microscopy images were obtained in a fluorescence phase microscope (Olympus Bx 51W1L, Olympus Optical, Tokyo, Japan) with water immersion lens coupled to an imaging detection system (QImaging Retiga 13001 CCD, cooled monochromatic digital camera 12-bit, QImaging Burnaby, Canada). Images were captured with Metafluor software (Universal Imaging Corp., PA, USA). To provide a sufficient number of frames for proper basal conditions estimation, at least 10 seconds were captured before the mechanical stimulus at each experiment. Experiments where stopped when the operator could not see any significant intensity change in the ratio image displayed by the Metafluor software.

## Results and discussion

In this section we show the results for the intermediate process (data calibration) as well as the final outputs (global analysis, local analysis and one filter computations). We processed a total of 22 experiments, from three main groups: 5 control, 5 with leptin and 12 with adiponectin, as described in the previous section.

### Data calibration

#### Statistical output

**Figure 7:**
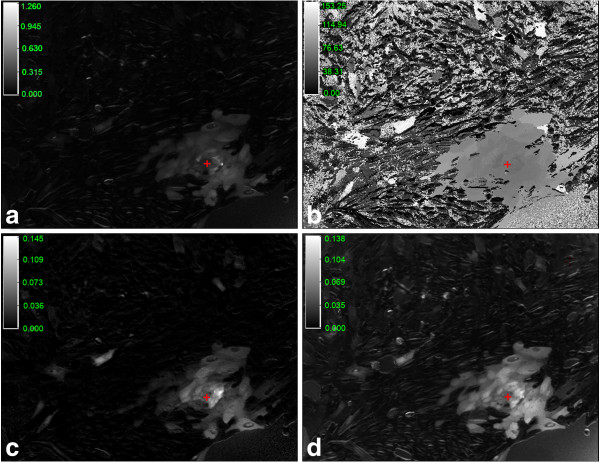
**Statistical information from the calibration process. a**) Maximum intensity at each pixel. **b**) Time when the maximum was found. **c**) and d) display the average intensity and standard deviation, respectively. Images were displayed with different scaling factors: (**a**) 1.26 [340/380 intensities], (**b**) 153.25 [s] (black is the beginning of the experiment, and white the end of it), (**c**) 0.144 [340/380 intensities] and (**d**) 0.138 [340/380 intensities]. Red crosses indicate the location of the mechanical stimulation.

Figure 7 shows the parameters that can be extracted, pixel by pixel, after the basal conditions were subtracted: (a) maximum intensity, (b) time at maximum intensity (zero is the beginning of the experiment, and white the end of it), (c) average and (d) standard deviation of intensities.

#### Execution times

On an Intel Core 2 Quad, 2.4Ghz processor, and 4Gb of RAM, the whole process took in average 121.8 s (125 couples of frames, 340 and 380 nm) for the control group. Leptin and adiponectin examples took in average 106.3 s (121 couples of frames) and 83.4 s (101 couples of frames), respectively.

The most time consuming task is the image registration, followed by the readout and writing operations of the frames. Thus, the total execution time has a strong dependency on the number of images.

### Global analysis

#### Automatic calculation of the propagation origin

**Figure 8:**
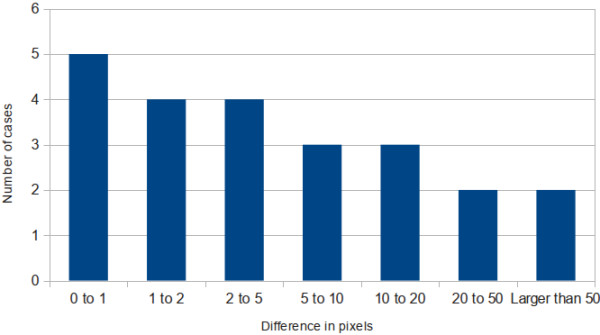
Histogram of the differences (measured in pixels) between automatic and manual location of the center of propagation.

We compared the results of the automatic calculation versus manual estimations done by four users. The differences between those measurements, calculated in pixels, were sorted and arranged in a histogram (Figure 8).

In 59% of the experiments, the automatic estimator yielded results closer than 5 pixels to the manual estimation. In less than the 20% of the cases, the difference was greater than 20 pixels. In those experiments, the automatic determination of the source failed because of corrupted data (miss-aligned frames), or due to spontaneous calcium releases in isolated cells in the tissue. Additionally, in 73% of the cases, users chose the automatic estimation as the correct place compared to the manual estimation.

#### Characterization of calcium wave velocities

**Figure 9:**
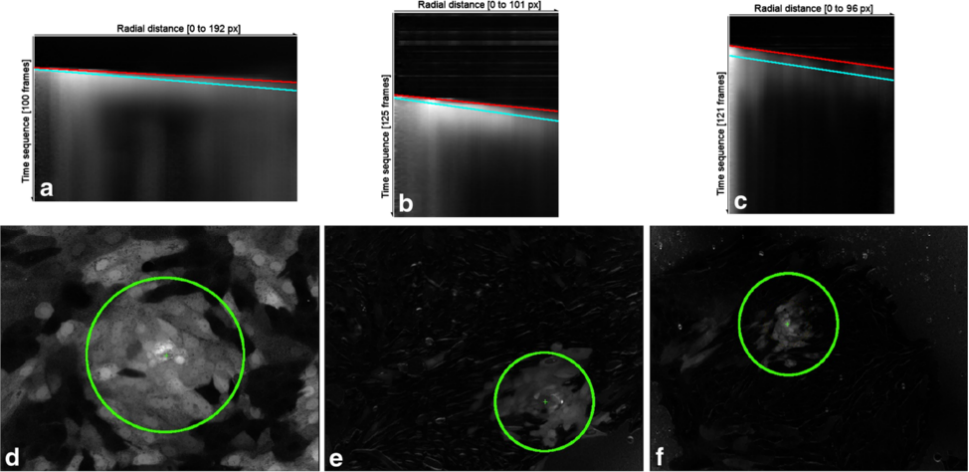
**Linear fits to maximum intensity (*cyan*) and maximum positive difference (*red*).** Slopes give the mean velocity of these measurements. **a**) corresponds to adiponectin (100 frames, 1.03 s/frame); **b**) to control (125 frames, 1.226 s/frame); and **c**) to leptin (121 frames, 1.233 s/frame) experiments. The corresponding radial distance considered for each experiment was: **d**) 192px, **e**) 101px and **f**) 96px. Scale: 1.025 um/px.

The propagation speeds were computed from a linear fit of the maximum value or of the largest increase in calcium level (Figure 9). Calculated velocities are in pixels/second and they can be transformed into physical units multiplying by the spatial resolution of our experiments (1.025 um/pixel).

**Figure 10:**
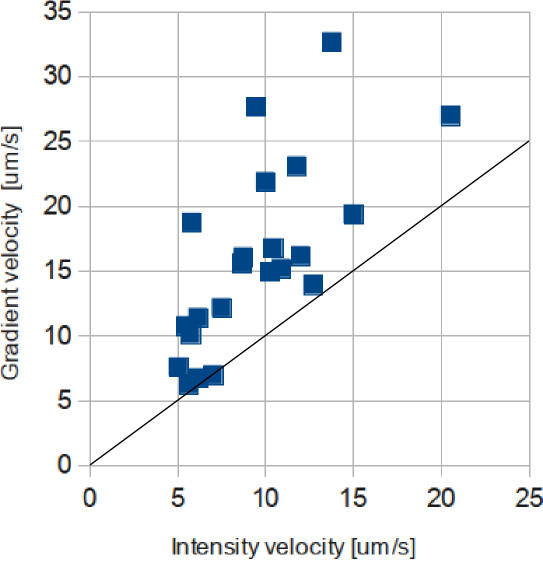
**Comparison between characterization of the speed of the wave using maximum intensities and maximum gradients.** 22 experiments.

The velocities computed from maximum gradients were larger than those computed from maximum intensities (Figure 10), except in one case, where noise and artifacts derived from misregistration of frames made the maximum intensity measurement a poor indicator of the wave velocity. Mean ratio factor (maximum intensity velocity over maximum gradient velocity) was 0.64, with standard deviation of 0.19. As the radius increases, it is expected that the dispersion of the cell ignitions increase. This is reflected by a growing separation between the maximum intensities and the maximum gradients, and thus, velocities derived from maximum gradients should be always larger than those derived from maximum intensities. Otherwise, it is an indication of a problem in the data extraction.

**Figure 11:**
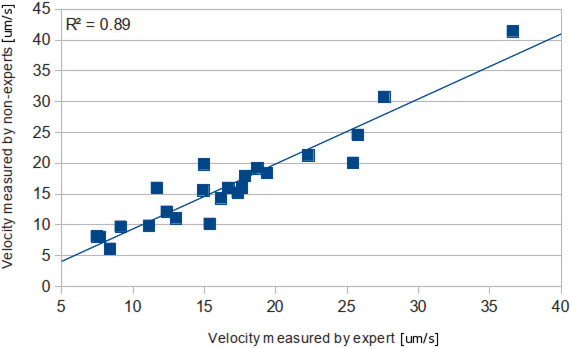
Experiments processed independently by experts and non-experts.

Reproducibility and robustness of this procedure were also tested. Non-expert users reprocessed a set of experiments, measuring the maximum gradient velocity without masks. Users had to determinate the source of the wave propagation and the distance to which intensities are used to calculate mean velocities (Figure 11). R^2^ of the trend line was 0.89.

#### Execution time

Execution times in the three kinds of experiments had a mean of 11.0 s for a maximum radius of 250 pixels. Reducing maximum radius has little impact on performance. For 125 frames, it took 11.63 s for a circular region with a radius of 250 pixels and 8.9 s for a radius of 100 pixels.

### Global analysis (function fitting)

#### Examples of results

**Figure 12:**
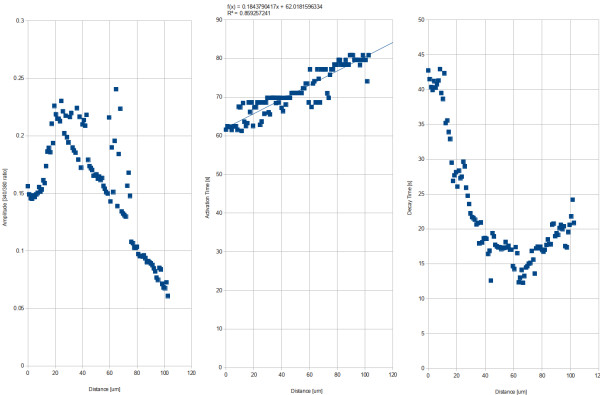
Parameter evolution for a control experiment, depending on the distance to the mechanical stimulus, in physical units.

A truncated exponential decay was fitted to the data, to see the evolution of the parameters as a function of radius. Figure 12 shows the amplitude (intensity at the activation time), the activation time, and the decay rate.

A simple linear regression to the activation time (Figure 12 b) gives another characterization of the wave propagation velocity. In this case, 5.1 pixels/s. Mean decay rate was 22.44 seconds.

#### Execution time

For a three-parameter function (simple exponential decay), and one hundred fitted functions (one for each radius), the process took in average 1.4 s.

### Local analysis (function fitting)

#### Examples of results

**Figure 13:**
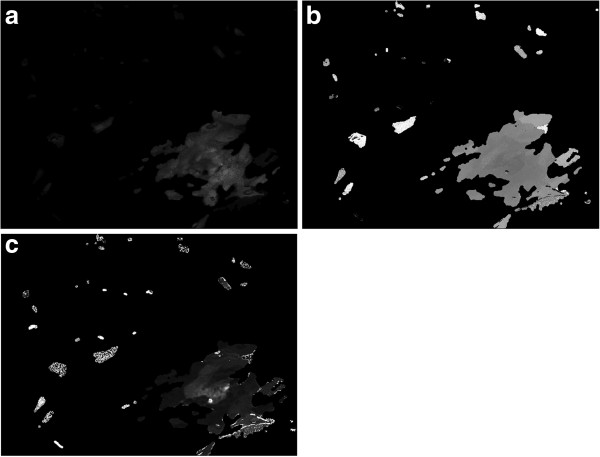
**Parameters obtained from the function fitting, inside a region of interest.** Non-processed pixels remain black. Control experiment. **a**) is the maximum amplitude of the wave, **b**) the activation time, and **c**) the decay rate time. Both time scales are normalized using the experiment’s duration. White pixels indicate the end of acquisition.

We used a simple exponential decay to create a map of each parameter, and see the variations along the whole experiment. Since the calculations are intensive and each image is mostly dominated by noise or by cells that were not activated, we use a mask to limit the optimization process to a region of interest (Figure 13).

#### Execution time

The image in the example was 650 × 500 pixels. A full frame optimization (fitting 325000 functions) took in average 666.3 s. Using the mask, the execution time went down to 273.6 s.

### One filter global analysis

#### Ring samples

**Figure 14:**
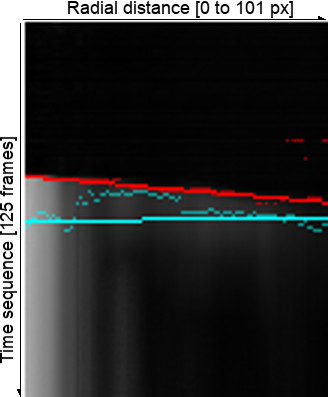
**Ring sample integration of 380 nm data.** This shows the average pixel intensities in time (*vertical axis*), for radial distances (*horizontal axis*). In cyan are shown the maximum intensity points and the best linear fit using outliers rejection. Shown in red are the points of maximum gradient, and the best linear fit.

As in the two-filter method, we built for every frame an image showing mean intensities for each ring sample with its linear fit of the maximum intensities and maximum gradients (Figure 14).

Since pure 380 nm data is not an indicator of the calcium concentration, maximum intensities are not correlated with the maximum concentration. This is especially true in the regions surrounding the mechanical stimulus.

#### Wave-front velocity

**Figure 15:**
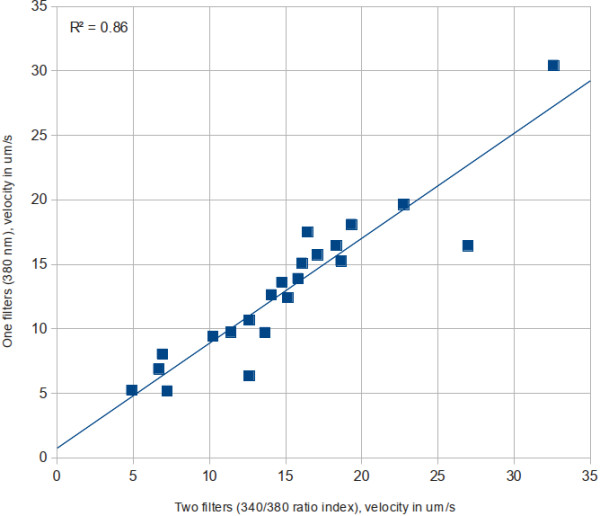
Comparison between velocities found with the two-filter imaging method, and using only the data from the 380 mm filter.

Characterization of the propagation of the calcium wave by the 380 nm maximum calcium increase enables us to determinate that velocity using only one filter. Figure 15 shows a comparison between velocities using one and two filters for our 22 experiments.

The correlation of the measured velocities was 0.93. The mean ratio factor between velocities (two filter’s velocity by one filter’s velocity) was 1.18, with a standard deviation of 0.25.

## Conclusions

The proposed data calibration process addressed some key factors that are commonly ignored or dismissed. Registration of frames ensures consistency between spatial locations, and yields better resolution in stacked images, as well as in the construction of the basal conditions. The natural decay of the Fura-2 marker must be considered to perform model fittings and comparisons. To achieve better calibration and basal conditions estimations, we suggest acquiring several frames before the mechanical stimulus. We used from 10 to 50 frames.

Noise management is another key feature of this calibration pipeline. Small median filters are effective to remove high variations (noise) in pixel values, while preserving boundaries and relevant features. Since the calcium concentration is derived from the ratio of two images, noise is not well correlated to intensities, and noise tends to be amplified.

Analysis of the calcium wave through a global approach simplifies the data, enables to extract a few parameters that characterize the process, and provides with a quick way to compare different groups or populations.

One of these parameters is the velocity of the traveling wave. Since we are assuming radial symmetry, it is of great importance to determine the origin of the wave. The proposed method of iterative calculations of the mass center retrieves a user independent point, with a high success rate (in our experiments, 57% showed a difference smaller than 5px to a manual estimation, and 83% showed a difference less than 20px). This increases reproducibility of the experiment, and reduces inter-operator sources of errors. As seen in Figure 4, frames usually have secondary calcium release sources, or high noise levels. Such factors prevent the use of a single step mass center calculation as a good estimator of the mechanical stimulus location. In this sense, our iterative approach detects the brightest and largest object, and then calculates its mass center efficiently.

Integration of one pixel-width ring samples gives a higher sampling rate than alternative methods, like averaging square samples on a matrix distribution, or over lines. Additionally, this method does not mix information at different distances, increasing spatial resolution and isolation. Manual extraction and integration of such amount of information would be unaffordable for a human operator.

We propose two methods to estimate the velocity of the wave, measuring the maximum intensity of the calcium concentration, and the maximum increase of the calcium levels, for every sampled radial distance. The wave-front is more related to the maximum gradient, since it detects the highest change in the calcium concentration. Analysis of the maximum concentration is affected by delays in the release of calcium by the cells, and homogenization of intra cellular levels. Increasing distances also produces dispersion of activation times, and hence it explains the lower measured velocities, compared to maximum gradient velocities.

Nevertheless, both velocities are highly correlated, and any of them characterizes the wave, enabling statistical analysis of different groups (in our case controls, leptin and adiponectin exposures). Our measurements are in agreement with previous experiments by other groups. Chopra et al. [13], reported intra-cellular velocities up to 100 um/s in cardiac myocytes. Sanderson et al. [2], working with epithelial cells in the respiratory tract, measured velocities in the cytoplasm of nearly 25 um/s. Since we are measuring mean propagation velocities, which are affected by delays between cells, lower results are expected (we obtained a mean propagation velocity of 15 um/s).

The optimization process proved to be a very efficient and powerful tool to retrieve deeper knowledge of the calcium wave. Our naive functions (truncated exponential decay, and square decay) yielded useful qualitative information. However, alternative fitting functions might be investigated to improve the match with the acquired data.

Global Analysis can be improved using signal masks. This way data are less corrupted by background noise, and mean intensities are more related to the values of the pixels of stimulated cells than those that have not been affected by the calcium wave. Since the number of non stimulated cells is not proportional to distance, or a linear function, there is not a simple way to correct this effect, rather than masking the signal by thresholding with a factor of the maximum measured intensity. Fittings should be done over masked data. When wave velocities are measured, masks give a better statistical significance to the study, since computations are done over data with high signal to noise ratio.

Masks are also helpful in the local analysis, since they restrict the number of analyzed pixels, and therefore execution times are lower. They also improve the visualization of the results.

Local analysis yields a set of parameters for each pixel. It might be difficult to take advantage quantitatively of such amount of information, but a qualitative inspection of maps containing those parameters yields a better understanding of the global analysis findings. Differences are not high between neighboring pixels in the same cell. There is a high correlation between the wave amplitude, time of activation, and the statistical output from the data calibration process (activation time is close to the time of the maximum intensity, and wave amplitude is similar to the maximum intensity). Feeding the optimization process with those values as starting points reduces the number of iterations, and hence the execution time, and they may also improve the robustness of the algorithm. Decay rates seem to diminish with distance for cells nearby to the mechanical stimulus, but after that it begins to raise again. Local analysis reveals that this behavior may be explained by low intensity, and noisy pixels. Further studies are needed to confirm this, and retrieve a model of the decay rates. Also, since the parameters seem to be cell-related (in terms of distance to the stimulus), large variations inside a cell are an indication of poor signal to noise, and results may lack of confidence. Nevertheless, propagation velocity of the wave may be derived only using the Global Analysis strategy, enhanced with a signal mask, so Local Analysis is left as a tool to be used when specific spatially varying information is needed.

Studies that are focused on measurements of the wave propagation velocity can be significantly improved capturing data from one filter, instead of the standard two-filter method. Data acquisition with two filters involves a mechanical rotation of a filter wheel, introducing a time delay between exposures. Time frequency is then limited by the speed of the mechanical system, exposure times, and properties of the camera and data transfer or storage. Using one filter, we are saving time, allowing a higher sampling rate, with the same exposure times. This increased frequency is needed to achieve a higher temporal resolution in the maximum increase of the calcium levels, used to characterize the wave-front.

Data from the 380 nm filter showed a strong correlation of the maximum gradient. Velocities measured by either method are equivalent. Maximum intensities in the 380 nm filter are not well correlated with the maximum calcium concentration; thus, measuring the wave velocity using this strategy was discouraged.

Execution time of the entire sequence may take up to half an hour, including visual inspection of the data to manually detect the frame where the wave propagation begins. User inputs may be reduced to a minimum set of parameters that are easily determined by inspection.

If no model fitting is needed, extracting only the propagation velocity by linear regression reduces the computation time to a few minutes.

Compared to manual methods, even the full process presents a considerable saving of time, with more reliable statistical results (from several hours, to just a few minutes). Considering computing time only, we need less than 15 minutes (data calibration, mask generation, global and local analysis). Manual work consists mainly of the necessary steps to set the processes’ parameters, like identifying the frame of the mechanical stimulation, or evaluating results and fine-tuning parameters (as in the signal mask creation, or the maximum distance used in the global analysis). In the current scheme, this manual workload may take from 5 minutes to 10 minutes, considering time consumed by the computer in non-optimal results. Since human supervision is desirable for optimal results, further automation should be emphasized on those steps that are required to set parameters that do not require fine-tuning, or are highly case dependent. The most time demanding tasks in that field are those related to inspection of the image series, or an animation of the frames.

From our experimental data, meaningful differences in propagation velocities were found between samples containing leptin, adiponectin and the control group, as presented in [12]. Nevertheless, further studies are needed to confirm that hypothesis, with a larger set of experiments and concentrations.

### Future work

Future development may be achieved modeling the calcium wave with diffusion equations, and taking advantage of the quantitative properties of the optimization process. This should also involve experimental modeling of parameters, as a function of radial distances.

The proposed one filter method should be used to measure fast traveling waves, and to compare behavior of different groups. Statistical confidence analysis of individuals would enable to study the effect of different hormones and proteins in calcium propagation, and in biological mechanisms.

Additional image processing techniques for automation may enable larger studies, and faster results, with less manual work and operators, as suggested in the previous subsection. Again, this should have a positive impact on statistical reliability of the studies.

In terms of automation, one step that might be further improved is the detection of the frame where the propagation begins. This is the first image were a large calcium release is detected, or major morphological changes of the stimulated cells are presented. We implemented a prototype automatic function to detect this, using the total flux plot. This strategy used a linear fit starting from the maximum calcium level to the previous frames, looking for the intersection of this line to the linear fit that described the basal conditions. In most cases, we achieved an accuracy of + −2 frames. This could be a good starting point for a more robust search, which may be based on the iterative center of mass calculation described in section 2.3.1, over the candidate frames. There, the last frame (in terms of acquisition time) may be used to create a signal mask, to avoid spurious data. Data may also be preprocessed by a soft-thresholding scheme, also described in 2.3.1, with a value that depends on the standard deviation of pixel values in the background. Since the calcium propagation is very symmetric at the beginning of the experiment, a large deviation of the mass center, and changes in intensity, might help in this detection.

Even though the current manual inspection is easy to perform and fast, signal masking might by further automated through the minimization of a “quality potential” with a two-step search. To optimize the threshold selection, a binary search may be used, expressing the threshold in terms of the median value of the image, and measuring amplitudes in standard deviations. Since the combinations of morphological operations are limited, an exhaustive search will take less than 5 seconds. The quality potential may compare the 2-norm differences between a low pass filtered version of the image, and the image multiplied by the mask.

Finally, automation of the selection of maximum distance to use in the Global Analysis may also be implemented, using two criteria: local dispersion of the maximum intensity or maximum gradient point, and a signal to noise ratio estimation for each radial distance. This strategy should reject too large distances, where linearity is lost, or no meaningful data was extracted, and include as many samples as possible, to ensure good statistics and consistency. This is especially true for a two-filter method, where frequency sampling is limited, and thus, larger distances are needed for accurate measurements.

## Competing interests

The authors declare that they have no competing interests.

## Authors’ contributions

CM was the main developer of the pipeline and software and main writer of the paper. CO and MV captured the experimental data and performed manual measurements. SU helped in the pipeline development and readproofing. CL and CP developed the non linear minimization strategy and readproofed. MA and PI developed the image processing strategies and readproofed. CT supervised the pipeline and software development, and cowrited the paper. All authors read and approved the final manuscript.
